# The genome sequence of the Ruby Tiger, *Phragmatobia fuliginosa* (Linnaeus, 1758)

**DOI:** 10.12688/wellcomeopenres.19204.1

**Published:** 2023-03-20

**Authors:** Douglas Boyes, Owen T. Lewis, University of Oxford and Wytham Woods Genome Acquisition Lab, Darwin Tree of Life Barcoding collective, Wellcome Sanger Institute Tree of Life programme, Wellcome Sanger Institute Scientific Operations: DNA Pipelines collective, Tree of Life Core Informatics collective, Darwin Tree of Life Consortium

**Affiliations:** 1 UK Centre for Ecology & Hydrology, Wallingford, England, UK; 2 Department of Biology, University of Oxford, Oxford, England, UK

**Keywords:** Phragmatobia fuliginosa, the Ruby Tiger, genome sequence, chromosomal, Lepidoptera

## Abstract

We present a genome assembly from an individual male *Phragmatobia fuliginosa* (the Ruby Tiger; Arthropoda; Insecta; Lepidoptera; Erebidae). The genome sequence is 629.4 megabases in span. Most of the assembly is scaffolded into 28 chromosomal pseudomolecules, including the assembled Z sex chromosome. The mitochondrial genome has also been assembled and is 15.4 kilobases in length. Gene annotation of this assembly on Ensembl identified 13,338 protein coding genes.

## Species taxonomy

Eukaryota; Metazoa; Ecdysozoa; Arthropoda; Hexapoda; Insecta; Pterygota; Neoptera; Endopterygota; Lepidoptera; Glossata; Ditrysia; Noctuoidea; Erebidae; Arctiinae; *Phragmatobia*; *Phragmatobia fuliginosa* (Linnaeus, 1758) (NCBI:txid214311).

## Background

The ruby tiger *Phragmatobia fuliginosa* is a distinctive moth in the subfamily Arctiiinae, the only representative of its genus recorded in the UK. In southern Britain, adult moths have pinkish-red or pinkish-brown forewings and mostly bright pink hindwings that are usually hidden when the moth is settled. Moths from northern Britain are generally darker and have been placed in the subspecies *borealis* (Staudinger) ( Waring *et al.*, 2017).

*Phragmatobia fuliginosa* has a range that extends across much of Europe and Asia, as well as parts of northern North America ( GBIF Secretariat, 2022). It has a wide distribution in Great Britain and Ireland, occurring mostly in in open habitats, and is absent only from Shetland. Adults are occasionally active during the day but are more likely to be recorded at light ( South, 1961). In northern Britain there is typically a single annual generation ( Waring *et al.*, 2017), but in southern Britain there are usually two generations, with adult moths recorded in small numbers from April until June, and in much higher numbers during July and August ( Randle *et al.*, 2019). The apparent high abundance of the second generation relative to the first may in part result from the late summer generation being more attracted to light traps ( Waring *et al.*, 2017).

The spherical white eggs of *P. fuliginosa* are deposited in batches, and the larvae are polyphagous, consuming a wide variety of mostly herbaceous plants, with a particular fondness for ragworts ( *Senecio* spp.) ( Henwood *et al.*, 2020). The hairy larvae overwinter fully-grown. South (1961) comments that “the vitality of caterpillars is extraordinary”, reporting an observation of a larva that was embedded in ice for at least 14 days without apparent harm. In the spring, the dark-coloured larvae bask in sunshine to raise their body temperature well above ambient, and the speedy larvae are often observed crossing roads and paths.

Male pheromones used in *P. fuliginosa* courtship are derived from pyrrolizidine alkaloids (PAs) obtained during larval feeding ( Krasnoff & Roelofs, 1990). A genome sequence for *Phragmatobia fuliginosa* will facilitate studies into molecular adaptations to polyphagy, the evolution of pheromone-based courtship, and contribute to a growing data set of resources for understanding lepidopteran biology more widely.

The genome of *Phragmatobia fuliginosa* was sequenced as part of the Darwin Tree of Life Project, a collaborative effort to sequence all named eukaryotic species in the Atlantic Archipelago of Britain and Ireland. Here we present a chromosomally complete genome sequence for *Phragmatobia fuliginosa*, based on one male specimen from Wytham Woods, Oxfordshire, UK.

### Genome sequence report

**Figure 1.**
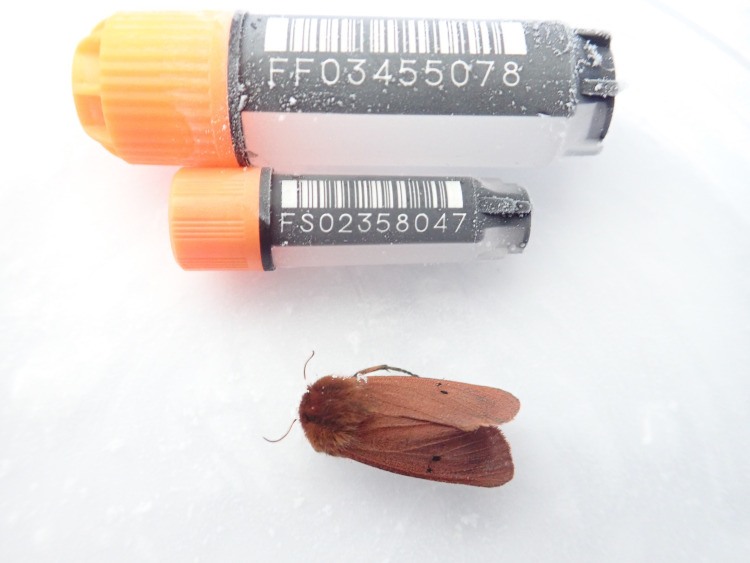
Photograph of the *Phragmatobia fuliginosa* (ilPhrFuli1) specimen used for genome sequencing.

The genome was sequenced from one male *Phragmatobia fuliginosa* specimen ( Figure 1) collected from Wytham Woods, Oxfordshire, UK (latitude 51.77, longitude –1.34). A total of 35-fold coverage in Pacific Biosciences single-molecule HiFi long was generated. Primary assembly contigs were scaffolded with chromosome conformation Hi-C data. Manual assembly curation corrected 29 missing joins or mis-joins and removed seven haplotypic duplications, reducing the assembly length by 2.82% and the scaffold number by 15.79%, and decreasing the scaffold N50 by 2.33%.

**Figure 2.**
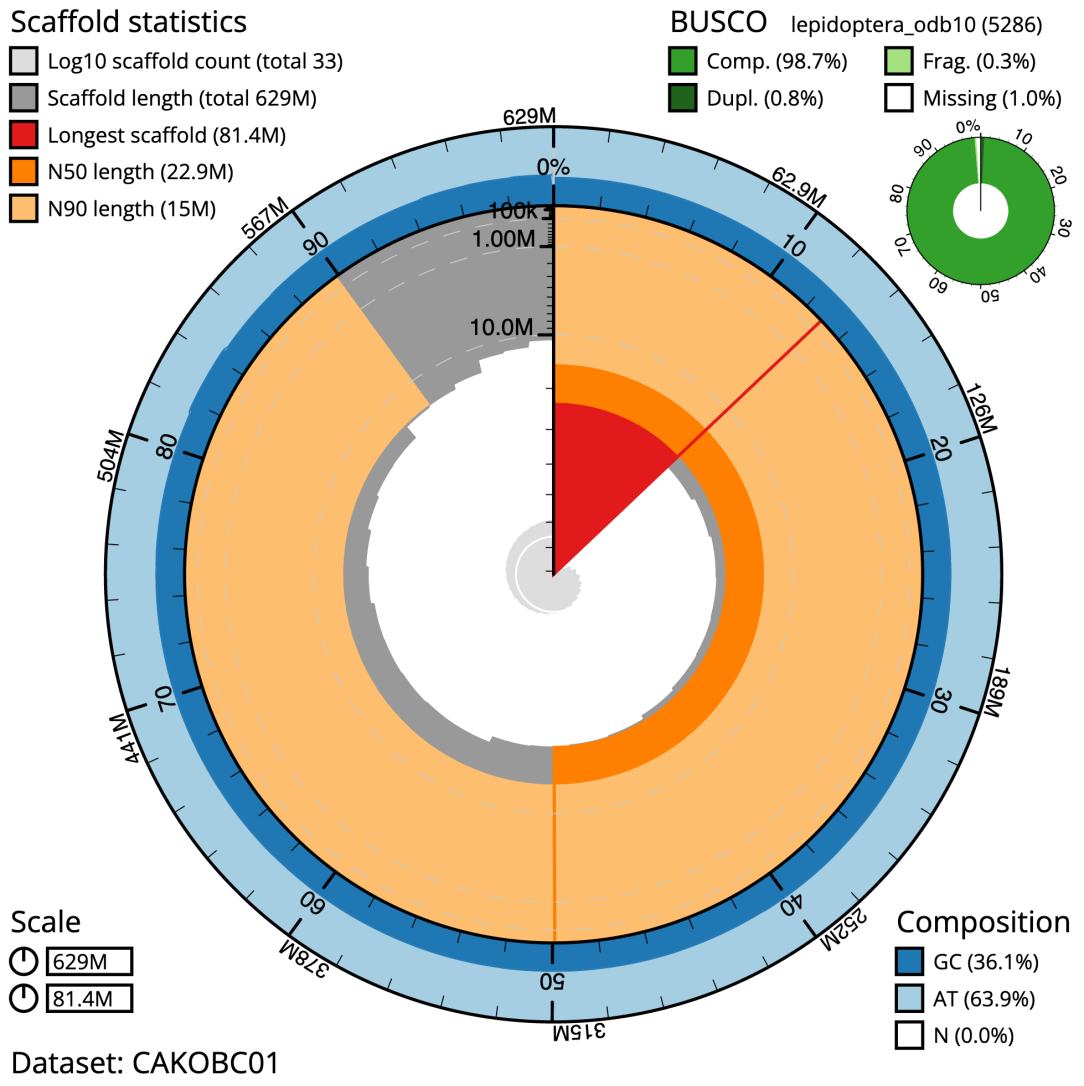
Genome assembly of *Phragmatobia fuliginosa*, ilPhrFuli1.1: metrics. The BlobToolKit Snailplot shows N50 metrics and BUSCO gene completeness. The main plot is divided into 1,000 size-ordered bins around the circumference with each bin representing 0.1% of the 629,457,366 bp assembly. The distribution of scaffold lengths is shown in dark grey with the plot radius scaled to the longest scaffold present in the assembly (81,383,725 bp, shown in red). Orange and pale-orange arcs show the N50 and N90 scaffold lengths (22,865,098 and 15,039,256 bp), respectively. The pale grey spiral shows the cumulative scaffold count on a log scale with white scale lines showing successive orders of magnitude. The blue and pale-blue area around the outside of the plot shows the distribution of GC, AT and N percentages in the same bins as the inner plot. A summary of complete, fragmented, duplicated and missing BUSCO genes in the lepidoptera_odb10 set is shown in the top right. An interactive version of this figure is available at https://blobtoolkit.genomehubs.org/view/ilPhrFuli1.1/dataset/CAKOBC01/snail.

The final assembly has a total length of 629.4 Mb in 32 sequence scaffolds with a scaffold N50 of 22.9 Mb ( Table 1). Most (99.97%) of the assembly sequence was assigned to 28 chromosomal-level scaffolds, representing 27 autosomes, and the Z sex chromosome. Chromosome-scale scaffolds confirmed by the Hi-C data are named in order of size ( Figure 2– Figure 5; Table 2). The assembly has a BUSCO v5.3.2 ( Manni *et al.*, 2021) completeness of 98.7% (single 97.9%, duplicated 0.8%) using the lepidoptera_odb10 reference set. While not fully phased, the assembly deposited is of one haplotype. Contigs corresponding to the second haplotype have also been deposited.

**Table 1.** Genome data for *Phragmatobia fuliginosa*, ilPhrFuli1.1.

Project accession data		
Assembly identifier	ilPhrFuli1.1	
Species	*Phragmatobia fuliginosa*	
Specimen	ilPhrFuli1	
NCBI taxonomy ID	214311	
BioProject	PRJEB50747	
BioSample ID	SAMEA7701498	
Isolate information	ilPhrFuli1: male, abdomen (DNA sequencing); head and thorax (Hi-C scaffolding)	
Assembly metrics *		*Benchmark*
Consensus quality (QV)	66.8	*≥ 50*
*k*-mer completeness	100%	*≥ 95%*
BUSCO **	C:98.7%[S:97.9%,D:0.8%], F:0.3%,M:1.0%,n:5,286	*C ≥ 95%*
Percentage of assembly mapped to chromosomes	99.97%	*≥ 95%*
Sex chromosomes	Z chromosomes	*localised homologous pairs*
Organelles	Mitochondrial genome assembled.	*complete single alleles*
Raw data accessions		
PacificBiosciences SEQUEL II	ERR8575386	
Hi-C Illumina	ERR8571673	
Genome assembly		
Assembly accession	GCA_932526445.1	
*Accession of alternate haplotype*	GCA_932526455.1	
Span (Mb)	629.4	
Number of contigs	73	
Contig N50 length (Mb)	14.1	
Number of scaffolds	32	
Scaffold N50 length (Mb)	22.9	
Longest scaffold (Mb)	81.4	
Genome annotation		
Number of protein-coding genes	13,338	
Number of non-coding genes	2,396	
Number of gene transcripts	22,406	

* Assembly metric benchmarks are adapted from column VGP-2020 of “Table 1: Proposed standards and metrics for defining genome assembly quality” from ( Rhie *et al.*, 2021). ** BUSCO scores based on the lepidoptera_odb10 BUSCO set using v5.3.2. C = complete [S = single copy, D = duplicated], F = fragmented, M = missing, n = number of orthologues in comparison. A full set of BUSCO scores is available at https://blobtoolkit.genomehubs.org/view/ilPhrFuli1.1/dataset/CAKOBC01/busco.

**Figure 3.**
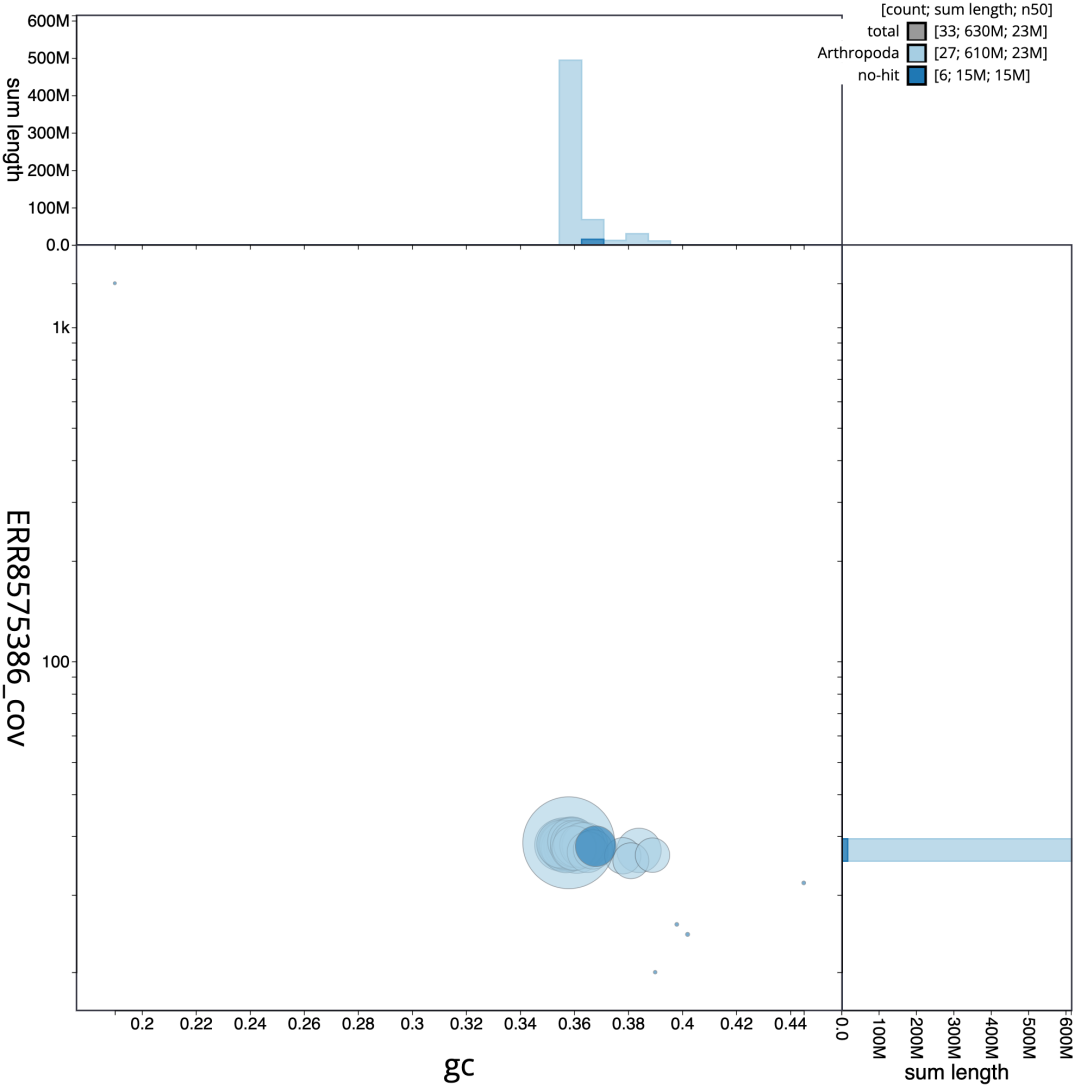
Genome assembly of *Phragmatobia fuliginosa*, ilPhrFuli1.1: GC coverage. BlobToolKit GC-coverage plot. Scaffolds are coloured by phylum. Circles are sized in proportion to scaffold length. Histograms show the distribution of scaffold length sum along each axis. An interactive version of this figure is available at https://blobtoolkit.genomehubs.org/view/ilPhrFuli1.1/dataset/CAKOBC01/blob.

**Figure 4.**
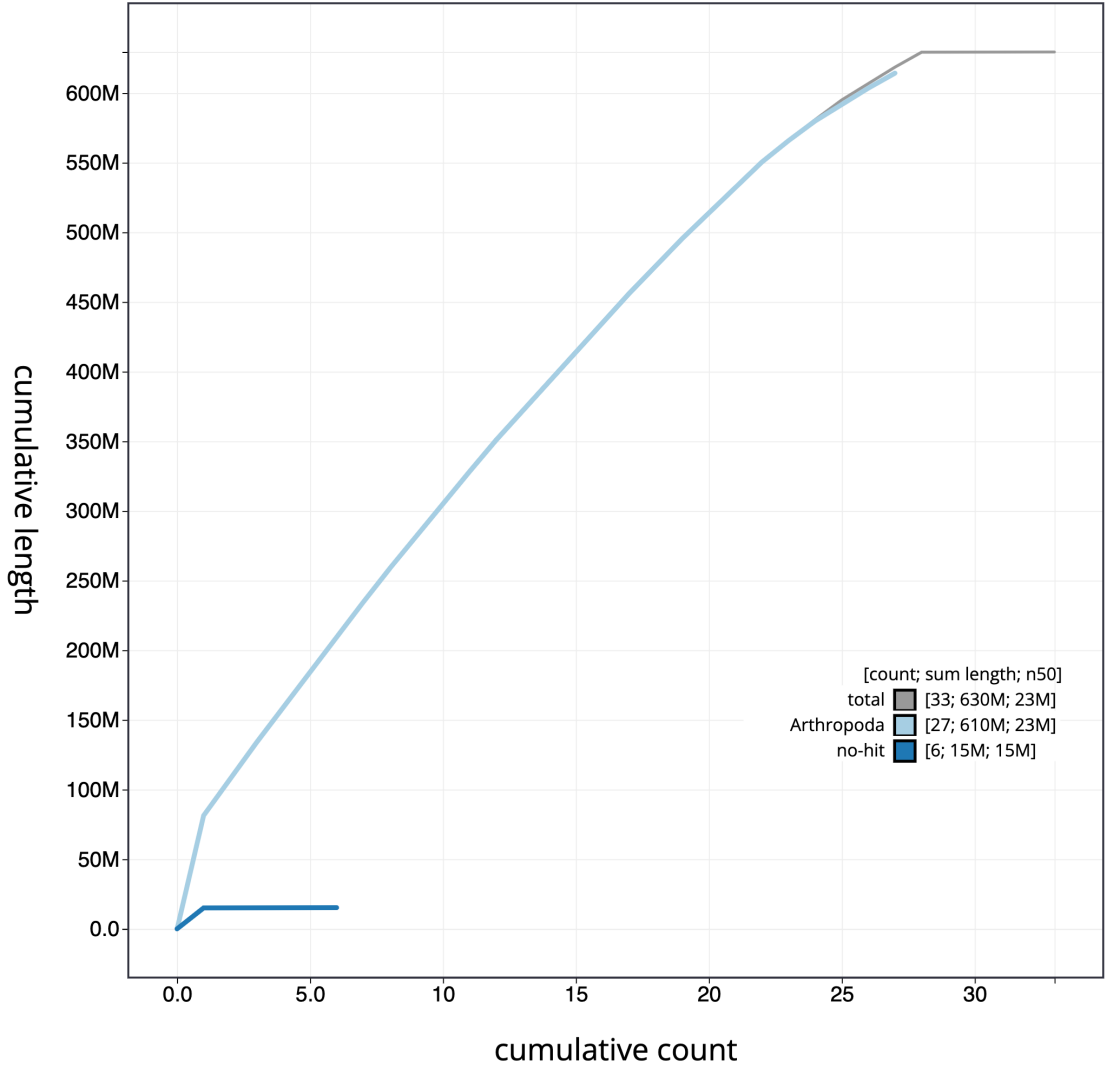
Genome assembly of *Phragmatobia fuliginosa*, ilPhrFuli1.1: cumulative sequence. BlobToolKit cumulative sequence plot. The grey line shows cumulative length for all scaffolds. Coloured lines show cumulative lengths of scaffolds assigned to each phylum using the buscogenes taxrule. An interactive version of this figure is available at https://blobtoolkit.genomehubs.org/view/ilPhrFuli1.1/dataset/CAKOBC01/cumulative.

**Figure 5.**
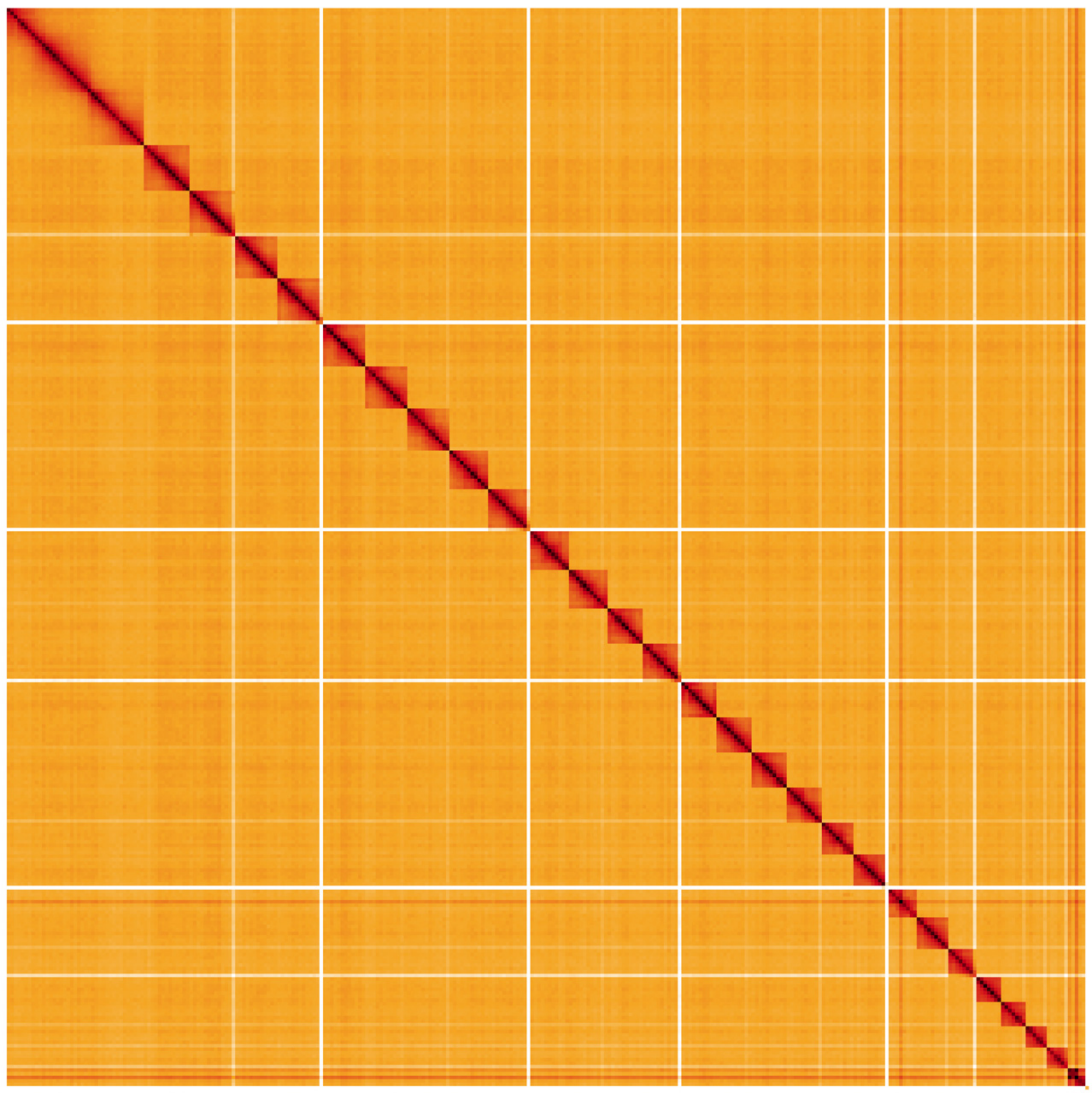
Genome assembly of *Phragmatobia fuliginosa*, ilPhrFuli1.1: Hi-C contact map. Hi-C contact map of the ilPhrFuli1.1 assembly, visualised using HiGlass. Chromosomes are shown in order of size from left to right and top to bottom. An interactive version of this figure may be viewed at https://genome-note-higlass.tol.sanger.ac.uk/l/?d=Cc6lMkieRfalxi0HQfR6Xw.

**Table 2.** Chromosomal pseudomolecules in the genome assembly of *Phragmatobia fuliginosa*, ilPhrFuli1.

INSDC accession	Chromosome	Size (Mb)	GC%
OW052058.1	1	26.74	35.9
OW052059.1	2	25.9	35.9
OW052060.1	3	25.37	35.6
OW052061.1	4	25.2	35.5
OW052062.1	5	25.01	35.8
OW052063.1	6	24.85	35.7
OW052064.1	7	24.31	36.1
OW052065.1	8	23.41	35.6
OW052066.1	9	23.17	35.8
OW052067.1	10	22.87	35.9
OW052068.1	11	22.68	35.7
OW052069.1	12	21.22	35.6
OW052070.1	13	21.2	35.5
OW052071.1	14	21.01	35.9
OW052072.1	15	20.88	36
OW052073.1	16	20.82	36
OW052074.1	17	20.03	36.1
OW052075.1	18	19.47	36.4
OW052076.1	19	18.72	36.3
OW052077.1	20	18.18	38.4
OW052078.1	21	18.09	36
OW052079.1	22	15.35	36.5
OW052080.1	23	15.04	36.8
OW052081.1	24	14.14	36.7
OW052082.1	25	11.92	37.8
OW052083.1	26	11.65	38.1
OW052084.1	27	10.68	38.9
OW052057.1	Z	81.38	35.8
OW052085.1	MT	0.02	19.2

### Genome annotation report

The *P. fuliginosa* genome assembly GCA_932526445.1 (ilPhrFuli1.1) was annotated using the Ensembl rapid annotation pipeline ( Table 1; Ensembl accession number GCA_932526445.1). The resulting annotation includes 22,406 transcribed mRNAs from 13,338 protein-coding and 2,396 non-coding genes.

## Methods

### Sample acquisition and nucleic acid extraction

A male *P. fuliginosa* specimen (ilPhrFuli1) was collected from Wytham Woods, Oxfordshire (biological vice-county: Berkshire) (latitude 51.77, longitude –1.34) on 13 June 2020. The specimen was taken from woodland habitat by Douglas Boyes (University of Oxford) using a light trap. The specimen was identified by Douglas Boyes using field ID and preserved on dry ice.

DNA was extracted at the Tree of Life laboratory, Wellcome Sanger Institute. The ilPhrFuli1 sample was weighed and dissected on dry ice with tissue set aside for Hi-C sequencing. Abdomen tissue was cryogenically disrupted to a fine powder using a Covaris cryoPREP Automated Dry Pulveriser, receiving multiple impacts. High molecular weight (HMW) DNA was extracted using the Qiagen MagAttract HMW DNA extraction kit. HMW DNA was sheared into an average fragment size of 12–20 kb in a Megaruptor 3 system with speed setting 30. Sheared DNA was purified by solid-phase reversible immobilisation using AMPure PB beads with a 1.8X ratio of beads to sample to remove the shorter fragments and concentrate the DNA sample. The concentration of the sheared and purified DNA was assessed using a Nanodrop spectrophotometer and Qubit Fluorometer and Qubit dsDNA High Sensitivity Assay kit. Fragment size distribution was evaluated by running the sample on the FemtoPulse system.

### Sequencing

Pacific Biosciences HiFi circular consensus DNA sequencing libraries were constructed according to the manufacturers’ instructions. DNA sequencing was performed by the Scientific Operations core at the WSI on Pacific Biosciences SEQUEL II (HiFi) instrument. Hi-C data were also generated from head and thorax tissue of ilPhrFuli1 using the Arima v2 kit and sequenced on the Illumina NovaSeq 6000 instrument.

### Genome assembly

Assembly was carried out with Hifiasm ( Cheng *et al.*, 2021) and haplotypic duplication was identified and removed with purge_dups ( Guan *et al.*, 2020). The assembly was then scaffolded with Hi-C data ( Rao *et al.*, 2014) using YaHS ( Zhou *et al.*, 2023). The assembly was checked for contamination and corrected as described previously ( Howe *et al.*, 2021). Manual curation was performed using HiGlass ( Kerpedjiev *et al.*, 2018) and Pretext ( Harry, 2022). The mitochondrial genome was assembled using MitoHiFi ( Uliano-Silva *et al.*, 2022), which performed annotation using MitoFinder ( Allio *et al.*, 2020). The genome was analysed, and BUSCO scores were generated within the BlobToolKit environment ( Challis *et al.*, 2020). Table 3 contains a list of all software tool versions used, where appropriate.

**Table 3.** Software tools and versions used.

Software tool	Version	Source
BlobToolKit	4.0.7	Challis *et al.*, 2020
Hifiasm	0.16.1-r375	Cheng *et al.*, 2021
HiGlass	1.11.6	Kerpedjiev *et al.*, 2018
MitoHiFi	2	Uliano-Silva *et al.*, 2022
PretextView	0.2	Harry, 2022
purge_dups	1.2.3	Guan *et al.*, 2020
YaHS	yahs-1.1.91eebc2	Zhou *et al.*, 2023

### Genome annotation

The Ensembl gene annotation system ( Aken *et al.*, 2016) was used to generate annotation for the *Phragmatobia fuliginosa* assembly (GCA_932526445.1). Annotation was created primarily through alignment of transcriptomic data to the genome, with gap filling via protein-to-genome alignments of a select set of proteins from UniProt ( UniProt Consortium, 2019).

### Ethics and compliance issues

The materials that have contributed to this genome note have been supplied by a Darwin Tree of Life Partner. The submission of materials by a Darwin Tree of Life Partner is subject to the Darwin Tree of Life Project Sampling Code of Practice. By agreeing with and signing up to the Sampling Code of Practice, the Darwin Tree of Life Partner agrees they will meet the legal and ethical requirements and standards set out within this document in respect of all samples acquired for, and supplied to, the Darwin Tree of Life Project. All efforts are undertaken to minimise the suffering of animals used for sequencing. Each transfer of samples is further undertaken according to a Research Collaboration Agreement or Material Transfer Agreement entered into by the Darwin Tree of Life Partner, Genome Research Limited (operating as the Wellcome Sanger Institute), and in some circumstances other Darwin Tree of Life collaborators.

## Data Availability

European Nucleotide Archive: *Phragmatobia fuliginosa* (ruby tiger). Accession number PRJEB50747, https://identifiers.org/ena.embl/PRJEB50747 ( Wellcome Sanger Institute, 2022) The genome sequence is released openly for reuse. The *Phragmatobia fuliginosa* genome sequencing initiative is part of the Darwin Tree of Life (DToL) project. All raw sequence data and the assembly have been deposited in INSDC databases. Raw data and assembly accession identifiers are reported in Table 1.

## Author information

Members of the University of Oxford and Wytham Woods Genome Acquisition Lab are listed here: https://doi.org/10.5281/zenodo.4789928.

Members of the Darwin Tree of Life Barcoding collective are listed here: https://doi.org/10.5281/zenodo.4893703.

Members of the Wellcome Sanger Institute Tree of Life programme are listed here: https://doi.org/10.5281/zenodo.4783585.

Members of Wellcome Sanger Institute Scientific Operations: DNA Pipelines collective are listed here: https://doi.org/10.5281/zenodo.4790455.

Members of the Tree of Life Core Informatics collective are listed here: https://doi.org/10.5281/zenodo.5013541.

Members of the Darwin Tree of Life Consortium are listed here: https://doi.org/10.5281/zenodo.4783558.
