# Supplementary material for: The genome sequence of the Ruby Tiger, Phragmatobia fuliginosa (Linnaeus, 1758)
Source: Wellcome Open Res. 2023 Mar 20;8:124. [Version 1] doi: 10.12688/wellcomeopenres.19204.1 (PMC11362730; doi:10.12688/wellcomeopenres.19204.1)
Supplement: Peer review history

# Reviewer report: Reviewer response for version 1

**DOI:** 10.21956/wellcomeopenres.21286.r58915

**Published:** 2024-08-29

**Authors:** Lapo Ragionieri

**Affiliations:** 1 Institute of Zoology, University of Cologne, Cologne, Germany

The manuscript entitled “The genome sequence of the Ruby Tiger, Phragmatobia fuliginosa (Linnaeus, 1758)” is well written and describes the genome of Phragmatobia fuliginosa. The authors utilized long-read sequencing technology (PacBio) to produce an initial assembly that was subsequently scaffolded using Hi-C, resulting in a genome assembly at the chromosome level with nearly all scaffolds assigned to chromosomes. All the information are clearly presented and the aim of the paper is to provide useful information to further studies about polyphagy, pheromone and Lepidopteran biology. The methods are appropriate, I only wonder if the authors can add few additional information.

 Below are some more specific comments and suggestions:

**Keywords:** The keywords need to be different from the title otherwise are useless.

**Species identification: **the author should cite the literature used.

**Command lines**: It is common practice to include the command lines used with all software. I believe this information can be very useful for the readers.

Are sufficient details of methods and materials provided to allow replication by others?

Partly

Is the rationale for creating the dataset(s) clearly described?

Yes

Are the datasets clearly presented in a useable and accessible format?

Yes

Are the protocols appropriate and is the work technically sound?

Yes

Reviewer Expertise:

Neuropeptidomics, Phylogeny, genomic

I confirm that I have read this submission and believe that I have an appropriate level of expertise to confirm that it is of an acceptable scientific standard.

## References

[1] : AUGUSTUS at EGASP: using EST, protein and genomic alignments for improved gene prediction in the human genome. *Genome Biol* .2006;7 Suppl 1(Suppl 1) : 10.1186/gb-2006-7-s1-s11 S11.1-8 10.1186/gb-2006-7-s1-s11 16925833 PMC1810548

# Reviewer report: Reviewer response for version 1

**DOI:** 10.21956/wellcomeopenres.21286.r65821

**Published:** 2023-09-06

**Authors:** Andrew Mongue

**Affiliations:** 1 Entomology and Nematology, University of Florida, Gainesville, Florida, USA

A concise and well-written example of a genome report. The manuscript is perfectly intelligible and suitable for indexing as is. In particular, I appreciate the table format for datasets, accessions, tools, and version numbers. I have only a couple of minor suggestions, ordered by importance:

- Chromosome number. The authors report n = 28 chromosomal scaffolds. The ancestral lepidopteran karyotype is thought to be n = 31, so this suggests a number of fusion events in the lineage leading to ruby tigers. I recognize it as beyond the scope of this paper to identify which syntenic units fused, but I believe it is worth mentioning as a point of future study, given the research interest in comparative lepidopteran genomics. 
- The background on life-history includes the phrase "the hairy larvae overwinter fully-grown". The phrasing is slightly ambiguous, as "fully-grown" often colloquially means "mature" or "adult", but I take the authors to mean that ruby tigers overwinter as last-instar caterpillars, as becomes apparent in the next sentence, but I still feel that "fully-grown" is a somewhat ambiguous term to use.

Are sufficient details of methods and materials provided to allow replication by others?

Yes

Is the rationale for creating the dataset(s) clearly described?

Yes

Are the datasets clearly presented in a useable and accessible format?

Yes

Are the protocols appropriate and is the work technically sound?

Yes

Reviewer Expertise:

Genome assembly, Lepidoptera, Hemiptera, evolutionary genetics, population genomics

I confirm that I have read this submission and believe that I have an appropriate level of expertise to confirm that it is of an acceptable scientific standard.

# Reviewer report: Reviewer response for version 1

**DOI:** 10.21956/wellcomeopenres.21286.r61044

**Published:** 2023-07-21

**Authors:** Scott Emrich

**Affiliations:** 1 University of Tennessee, Knoxville, TN, USA

Boyes and Lewis present a chromosome scale assembly of a a single male moth ( *Phragmatobia fulginosa*) native to Britain, which was sequenced as part of the Darwin Tree of Life Project. The primary reads were generated using PacBio HiFI and HiC was performed for chromosomal scaffolding. They were able to isolate the putative Z sex chromosome and the mitochondrial genome.

 Although the methods are sound and the assembly seems pretty typical given the species/technologies, I have three comments to aid in clarity for future readers and to put this paper in context. As such I have reservations as I would like these relatively small things to be addressed but also admit they are minor clarifications (#1 and #2) and addition of some more citations/context (#3), in large part since a big result I get out of this is you can use HiFi and HiC to assemble Lepidopteran genomes pretty well.

 First, there are no details in the manuscript how they determined which of the scaffolds was the Z scaffold. If this is because it is established in Leps that the largest scaffold is the sex chromosome they should add a citation, or if there are genetic markers that would help to assign the scaffolds to previously published linkage groups. I believe heterochromatic sex chromosomes was published in the early 1900s using this species ^1^ but not sure what types of more traditional genetics has been done since.

 Second, it is unclear from the text how the authors computed k-mer completeness presented in Table 1 since it isn't defined as a footnote and doesn't appear in other legends where BlobToolkit is referred to for the "traditional" assembly quality measures (e.g., Figure 2). Please make it clear how this was computed either as a footnote or in the methods.

 Finally, I don't expect any new biology in an open research note but the authors should put their work in context. For example, a PubMed search of "molecular adaptations to polyphagy" returns over 180 results, including crop pests that are probably better studied like fall army worm ^2^ and other Leps. Are there unique life history or other aspects that could be explored in the future, other than adding another Lep to (future) comparative genome analysis? Also, I know a lot of work on pheromones has been done in *Heliconius *(e.g., Byers *et al.*, 2021 ^3^ to cite a more recent one). Some citations and context would help clarify why this could be an important resource other than being one of the many genomes sequenced by the Darwin Tree of Life project.

Are sufficient details of methods and materials provided to allow replication by others?

Partly

Is the rationale for creating the dataset(s) clearly described?

Yes

Are the datasets clearly presented in a useable and accessible format?

Yes

Are the protocols appropriate and is the work technically sound?

Yes

Reviewer Expertise:

Genome assembly; next generation sequencing and its applications; arthropod genomics

I confirm that I have read this submission and believe that I have an appropriate level of expertise to confirm that it is of an acceptable scientific standard, however I have significant reservations, as outlined above.

## References

[1] : Geschlechtschromosomen bei Lepidoptere. *Zool. Anz.* .1913;41:246-251

[2] : Two genomes of highly polyphagous lepidopteran pests (Spodoptera frugiperda, Noctuidae) with different host-plant ranges. *Sci Rep* .2017;7(1) : 10.1038/s41598-017-10461-4 11816 10.1038/s41598-017-10461-4 28947760 PMC5613006

[3] : Clustering of loci controlling species differences in male chemical bouquets of sympatric Heliconius butterflies. *Ecol Evol* .2021;11(1) : 10.1002/ece3.6947 89-107 10.1002/ece3.6947 33437416 PMC7790645
